# Book Reviews

**Published:** 1916-04

**Authors:** 


### BOOK REVIEWS

**Books mentioned may be inspected at and ordered through this office. So far as possible, books received in any month will be reviewed in the issue of the second month following.**

---

**Reference Handbook of the Medical Sciences**; embracing the entire range of Scientific and Practical Medicine and Allied Sciences. By various writers. Third edition, completely revised and rewritten. Edited by Thomas Lathrop Stedman, A. M., M. D. Complete in eight imperial quarto volumes. Volume VI 967—6 double column pages, illustrated by 489 engravings and 7 full-page plates in black and colors. Wm. Wood & Co., New York.

The expense of this cyclopaedic work exceeds the entire capitalization of many publishing houses. Yet the success of the two earlier editions, justifies so large an investment. The first edition, published 1888, is still valuable for practical reference though, obviously the great advance in many lines of medical science and, indeed, the creation of entirely new lines of thought and action, reduces many articles to a historic use. The present volume extends from Ligation of arteries to Oxyuris. With the work three quarters completed, it is already available for practical reference and the remaining volumes will soon be issued so that those who have delayed subscribing, should do so promptly. While we would not go so far as to say that this work can adequately constitute an entire medical library, it comes close to this standard. A large corps of writers has co-operated in preparing articles, under the general guidance of Dr. Thomas L. Stedman. In each instance, the aim has been to secure a thorough discussion of a subject, omitting guess work and exploded theories, and condensing so far as possible, largely by a system of cross reference which will be expedited by the index. Thus, in the great majority of instances in which information is needed, the happy mean is secured between the use of a brief and incomplete text book and one so extensive as to burden the mind with unnecessary detail.

---

**Trachoma,** Its Prevalence, Effects upon Vision, and the Methods of Control and Eradication. Illustrated pamphlet, 40 pages, issued by the National Committee for the Prevention of Blindness, 130 E. 22, N. Y.

Although intended mainly as a means of popular education in regard to this serious and little realized danger, it is valuable to the medical profession, especially those of its members not directly interested in ophthalmology and who have not had experience in immigration inspection and have not studied the insidious progress of this disease in our country.

---

**Obstetrics.** A Practical Text Book for Students and Practitioners. By Edwin Bradford Cragin, A. B., A. M., (Hon.) M. D., F. R. C. S.; Professor of Obstetrics and Gynecology, College of Physicians and Surgeons, Columbia University, New York; Attending Obstetrician and Gynecologist to the Sloane Hospital for Women; Consulting Obstetrician to the City Maternity Hospital. Assisted by George H. Ryder, A. B., M. D.; Instructor in Gynecology, College of Physicians and Surgeons, Columbia University, New York; Assistant Attending Obstetrician, Sloane Hospital for Women; Associate Surgeon, Woman's Hospital, New York. Octavo, 858 pages, with 499 engravings and 13 plates. Cloth, $6.00 net.

Anatomy, embryology and physiology are well and thoroughly discussed and the further discussion, scientic and practical of pregnancy, includes about a quarter of the whole work before labor is reached. This is significant, not only as indicative of modern custom of regarding the pregnant woman as deserving special care, but because it is virtually a prophylaxis of many of the abnormalities later discussed. In fact, the entire work conceives of Obstetrics in a broad spirit and covers every detail of the management of the two lives at stake until they can safely be dismissed from the attendance of the physician in the role of obstetrician.

---

**New York Health Almanac.** Health News, the Monthly Bulletin of the N. Y. State Dept. of Health, takes the form of an almanac.

The ordinary tables of sun-rise and sun-set, moon's phases, morning and evening stars, etc., are given. Biographic and historic anniversaries are included, with a marked leaning toward those of medical importance. In place of the advice to take patent medicine which we have come to associate with almanacs, useful hygienic hints are given and even the jokes are represented by some very excellent wit. The Dept. of Health is to be congratulated in this answer to the question "Why should the marking of time and the presentation of astronomic events be relegated to quacks?" This almanac is well worth preservation and we trust that, at any rate after this year, the edition will be large enough so that anyone can secure it free, at a drug store, anywhere in the state. Its publication is a good deal more important in the history of our efficient Dept. of Health than might seem, from offhand consideration. In fact, with some further development, we consider it the best means of popular education in health matters that has been devised.

---

**The Mulford Laboratories.** The H. K. Mulford Co. of Philadelphia, has issued a collection of Lumiere color photographic reproductions (without retouching) showing their laboratories, drug farms, etc., with descriptive text.

This is interesting merely as an example of the beautiful achievements of this comparatively new art, still more so to the medical profession, in putting before them, the elaborate equipment of this progressive firm.

---

**A Practical Treatise on Infant Feeding and Allied Topics.** Harry Lowenburg, A. M., M. D., Philadelphia. F. A. Davis Co., Philadelphia. 382 pages, 64 text engravings, 30 original page plates, 11 in color. $3.00.

About half of the work is devoted to feeding, many of the plates in this section illustrating stools. The "allied topics"

include infantile atrophy, rickets, scurvy, vomiting, constipation, diarrhoea, spasmophilia, exudative diathesis, pyloric obstruction, etc. X-ray plates illustrate the principal mechanic abnormalities. The book is clearly written and the facts logically arranged. The tone of the work is practical throughout, all the more so because based on scientific principles, applied both in diagnosis and in giving a conception of each condition.

---

**Cancer of the Stomach.** A Clinical Study of 921 Operatively and Pathologically Demonstrated Cases, by Frank Smithies, M. D., Gastro-enterologist to Augustana Hospital, Chicago. With a Chapter on the Surgical Treatment of Gastric Cancer, by Albert J. Ochsner, M. D., Professor of Clinical Surgery in the University of Illinois. Octavo of 522 pages with 160 illustrations. Philadelphia and London: W. B. Saunders Company, 1916. Cloth, $5.50 net; Half Moroco, $7.00 net.

It cannot be too strongly emphasized that this work is mainly a collective clinical study of a large series of cases observed in such a way as to present practically complete information of each one, and that this series represents ten years or more of digestion of personal experience and study of the literature. While by no means an assembly of monographs, many of the items have been worked up in special articles published at various times. Ochsner's chapter on Surgical Treatment is, of course, authoritative and it is followed by a chapter on medical or, rather non-surgical treatment in which nothing that may influence a case favorably is ignored on account of prejudice, however justified. A few minor differences of opinion may be expressed: in titrating for free HCl, the end-point is not a straw yellow but that at which the cerise color disappears and gives place to an orange, the straw yellow including other acid factors, probably due to acid salts. We would go somewhat further than the statement that the alizarin test for combined acidity is of "dubious value" but, so long as it is mentioned at all, it should be stated that the combined acidity is determined by subtraction from the total acidity. The table of incidence according to occupation is interesting but we are not inclined to attach significance to the preponderance of farmers—32.4% and the occurrence of 11.3% more in farm-dwellers otherwise classified,—since 36% of the total population are engaged in agricultural pursuits. The careful, statistic studies of so-called specific reactions are of the utmost value. What impresses us most favorably is that, while the work is reduced to the basis of actual fact, so far as it can be determined for each detail, it does not upset preconceived ideas based on careful study, the authors are not hobbyists, do not go to extremes, they present their experience and convictions in a judicial spirit, make no plea for novelties and, in general, confirm rather than revolutionize the best opinions of the time.

---

**1916 Catalogue of the Latest Practical, Scientific, Technical and Automobile Books.**

Sent free on application to the Norman W. Henley Publishing Co., 132 Nassau St., N. Y.

---

**A Text-Book Upon the Pathogenic Bacteria and Protozoa. For Students of Medicine and Physicians.** By Joseph McFarland, M. D., Professor of Pathology and Bacteriology in the Medico-Chirurgical College, Philadelphia. Eighth edition, thoroughly revised. Octavo of 807 pages with 323 illustrations, a number of them in colors. Philadelphia and London: W. B. Saunders Company, 1915. Cloth, $4.00 net.

It gives us great pleasure to review this scholarly work of a classmate at the University of Pennsylvania. A little less than half of the book is devoted to general considerations of theory and technic though, even here, the practical element appears in the discussion of the preparation of vaccines, the bacteriologic analysis of air, water and foods, the determination of thermic death points and of the value of antiseptics and in the inclusion of the Wassermann test, illustrated in color. The following 40 chapters discuss in detail the diseases in which micro-organisms are known, the number of the latter being considerably greater as, for suppuration, pneumonia, typhoid, etc., various more or less specific or alternate germs are known. Blastomycosis and sporotrichosis are included. The Negri bodies for hydrophobia and the Bordet-Gengou bacillus for whooping cough are tentatively accepted. The discussion of micro-organisms by diseases, rather than by a biologic classification is in a sense, disappointing but it is highly practical, not so much because actual problems in bacteriologic diagnosis and therapy thus present themselves as because the attempt at accurate classification tempts from the field of fact into that of the imagination. A similar disappointment is encountered in the search for a description of the germs of the exanthemata, especially for the elaborate life-cycles of the variola-vaccinia protozoon, for the germ or germs of cancer, etc. In other words, the author has avoided the realm of speculation and contents himself with facts although, in several instances, micro-organisms not absolutely established as specific causes of disease but supported by good evidence, are considered, the existence of any reasonable doubt being carefully noted. The work is one that, undoubtedly, will receive additions in subsequent editions but there is little or nothing that will need correction. We need not recommend the book to laboratory workers; it is too well established by its reputation maintained and increased through eight editions but we wish that it might be carefully studied by every medical practitioner to afford a conception of the exact status of the science of bacteriology and, particularly, a knowledge of what is firmly established and a realization of what is merely aspiration, speculation or exploded theory.

---

**Urgent Surgery.** Felix Lejars, Paris, 3d English impression, translated from the 7th French edition by Wm. S. Dickie, F. R. C. S. and Ernest Ward, M. A., M. D., F. R. C. S. Vol. 2, G.-U. Organs, Rectum and Anus, Strangulated Hernias, Extremities. Wm. Wood & Co. 588 pages, 20 page plates and 1068 illustrations in the text. $7.

Mr. Dickie having been ordered to active military service, the translation has been continued by Dr. Ward, no break being apparent. Except incidentally, no attempt has been made to include strictly military surgery and, as noted in the title and stated in our previous review of the first volume, the discussion is limited, so far as possible, to strictly emergent operations.

---

**Principles and Practice of Physical Diagnosis.** By John C. DaCosta, Jr., M. D., Assistant Professor of Medicine, Jefferson Medical College, Philadelphia. Third edition, thoroughly revised. Octavo of 589 pages with 243 original illustrations. Philadelphia and London: W. B. Saunders Company, 1915. Cloth, $3.50 net.

What impresses us especially is the attention paid to inspection which is often given scant attention, and the detailed description of physical methods for particular clinical and pathologic conditions, even occasionally checked by post mortem findings. The sphygmograph and electro-cardiograph, fluoroscope and radiograph, tuberculin reaction, etc., come in for consideration but the work is held for the most part to establish limitations of the term physical diagnosis. We appreciate the mention of our studies of auscultatory percussion but think that the tuning fork substitute method should be included in the reference (Transactions of Med. Soc. State of N. Y. 1895). We are especially pleased to note that distinctions of pitch in percussion and auscultations were first developed by the original editor of this journal, Dr. Austin Flint, in 1852.

---

**Transactions of the 21st Annual Meeting of the American Laryngologic, Rhinologic and Otologic Society,** Chicago, June 15-16, 1915. Published for the Society by Paul B. Hoeber, N. Y., Geo. L. Richards, Fall River, Samuel J. Kopetzky, N. Y., and Frank E. Kittredge, publication committee.

In addition to lists of officers, fellows and transactions, a large number of papers on various subjects are included. One of the most generally interesting of these, is the prize essay of Gerhard H. Cocks of N. Y., conducted under the auspices of the N. Y. State Commission on Ventilation: Experimental Studies on the Effect of Various Atmospheric Conditions upon the Upper Respiratory Tract. These deal especially with the effects of changes of temperature and humidity. In the majority of instances, it was found that increase of temperature causes increased swelling, moisture and redness (measured chiefly by the turbinates), decrease the reverse, while moist heat increases the former reactions. Bacterial infection cannot entirely explain catarrhal inflammations. The problems are, however, complicated and the paper cannot be briefly abstracted.

---

**Autoplastic Bone Surgery.** By Charles Davison, M. D., Professor of Surgery and Clinical Surgery, University of Illinois, College of Medicine; Fellow of the American College of Surgeons; Surgeon to Cook County and University Hospital, Chicago, and Franklin D. Smith, M. D., Clinical Pathologist to University Hospital, Chicago. Octavo, 369 pages, with 174 illustrations. Cloth, $3.50 net.

The history of this subject is more ancient than is ordinarily supposed. It was in use for many centuries in the east and reached western Europe in the latter part of the 17th century. In 1682, Jobi Meekren, attempted to fill in a cranial defect in the skull of a soldier with bone from a dog but was compelled by the ecclesiastic authorities to remove it. Heteroplastic methods, which antedate the use of human bone grafts, are, however, less likely to be successful, quite apart from theologic objections. The recent history is epitomized and also reviewed in a bibliographic reference list. The various problems of histogenesis are carefully presented and special practical problems are discussed seriatim, with X-ray and photographic illustrations. In some instances, suggestions, not yet corroborated by actual experience or by any large series of cases, are recorded.

---

**The Art of Living Long.** Edited and published by Wm. F. Butler, Milwaukee. 214 pages.

This consists of a translation of Luigi Cornaro's La Vita Sobria, in four discourses written at the ages of 83, 86, 91 and 95, the total span of his life being from 1464 to 1566; of essays on the same general subject by Joseph Addison, Lord Bacon and Sir Wm. Temple; of a life of Cornaro and a genealogic review of his family; and of various minor notes and quotations. Cornaro was fortunately free from business worries but he states that his constitution was delicate and that he was originally of a choleric nature. He preaches abstemious habits in all respects but considered wine, especially at certain seasons, necessary to the preservation of his life. Tobacco, though known by the discoveries of Columbus and his successors during the latter part of Cornaro's life, is not mentioned. One of Cornaro's reasons for wishing a long life was the hope to see begun and completed, the protection of the lagoon on which Venice is situated—a desire quite in harmony with the spirit of modern preparedness. This book, though not strictly medical and indeed, scarcely adding to what is actually known regarding longevity, is most interesting and the editor and translator is entitled to the greatest credit.

---

**Memorable Addresses by American Patriots,** from collection of John Clark Ridpath, published by Elliott-Madison Co. Chicago and Jones Bros. Pub. Co., Cinti. 111 pages, paper.

This contains Patrick Henry's speech on Resistance to Oppression, Washington's Farewell Address, Lincoln's Gettysburg Address, Bryan's Cross of Gold, McKinley's International Amity, Taft's Philippines, etc.

---

**Colorado Industrial Plan,** John D. Rockefeller, Jr., concluding with the Agreement between the Colorado Fuel & Iron Co. and Employes. 95 pages, paper.

---

**Changes in the Food Supply and Their Relation to Nutrition.** Lafayette B. Mendel, New Haven, Yale University Press. 61 pages, $0.50.

Estimates based on increase of population, reserve arable lands and increase of productivity, indicate that the world will be up against the problem of getting enough to eat by the end of the present century. Proso millet and kaoliang are mentioned as cereals available for general use. Improvements in methods of preservation, transportation, etc., and the marked changes in dietetic customs, as the lessened use of meat and eggs, the great increase in the use of sugar, the more general availability of fresh vegetables or canned fruits, etc., in winter, are mentioned. While the author does not make this the main purpose of his thesis, it is clear that the limit of production to support the human race can be postponed quite a while beyond that calculated on present and past conditions.

---

**Handbook of Infant Feeding**, Lawrence T. Royster, M. D., Norfolk, Va., published by C. V. Mosby Co., St. Louis, 144 pages, $1.25.

This is an extremely practical, condensed treatise, covering both the general principles of infant feeding and the care of special nutritive conditions.

---

**Diagnostic Methods**, A Guide for History Taking, Routine Physical Examinations, etc., Herbert Henry Brooks, A. B., M. D.,. Memphis, Tenn., C. V. Mosby Co., St. Louis. 96 pages, illustrated, $1.00.

While a very useful guide for a college course, the scope of the book is obviously too limited for general use, although it cannot be denied that the purchaser will get his money's worth. It is admirably adapted to "brushing up," either for an examination or other purpose but we trust that the author, having demonstrated his grasp of the subject and his ability to present matter in readable form, will follow up this book with a more comprehensive treatise.

---

**Candy Medication, Bernard Fantus**, M. D., Chicago, published by C. V. Mosby Co., St. Louis. 82 pages, $1.00.

The surprisingly few officinal and proprietary preparations that are really pleasant, led the author to undertake various experiments toward the incorporation of medicaments with candy. Among the early attempts were a sulphur taffy and chocolate creams containing cod liver oil. (We recollect an unpleasant personal experience with such chocolate drops many years ago). A large number of available drugs are listed. Tablet machines and general principles of choice of excipient and flavors are described. 58 working formulae are given in detail, based on a list of 8 stock preparations. Among the latter, are found "Fat Starch," consisting of

15% of a 3% alcoholic solution of saccharin, 25% of liquid petrolatum (the purest forms should of course be used) and 75% of starch. The starch is mixed with the saccharin solution and, after complete evaporation of alcohol, the liquid petrolatum is incorporated. Cacao Sugar consists of 10% cacao powder, 10% dextrose (not absolutely necessary but making tablets of better consistence), 80 (or if dextrose is omitted 90) % powdered saccharose. This is flavored with ½ C.C. of 10% spirit of cinnamon per 100 grams. "Fat Sugars" consist of 20% of "Fat Starch" and 80% of powdered sugar, with small quantities of harmless dyes and flavors. Candy medication is a highly important advance not only in the therapeutics of children but of grown persons and is due very largely, almost entirely in its pharmaceutic technicalities, to the author.

---

**Starvation Treatment of Diabetes,** Lewis Webb Hill, M. D., and Rena S. Eckman, with introduction by Richard C. Cabot, Boston, published by W. M. Leonard, Boston, second edition, 131 pages.

Great credit is due to Dr. Allen for developing this system of treating diabetes although it is only fair to state that the term starvation is to be taken with qualification as to time and degree and the method, in its essentials, is practically identical with that which many have used who have understood the importance of acid intoxication, the danger of fat, the ultimate necessity of carbohydrates.

---

**Physiology of the Amino Acids.** Frank P. Underhill, Ph. D., New Haven, Yale University Press. 169 pages, illustrated, $1.35.

This is a critical review, summarizing the results of research in this branch of chemistry, with the needs of the clinician in mind. Experiments as to growth of experimental animals on different forms of proteid and their derivatives, are cited. It is impossible to do the work justice by abstracts. There are few branches of organic chemistry not directly applicable to diagnosis which have so important a practical bearing on medicine as this. The book should be read in its entirety. In relation to medical practice and in timeliness, it reminds us more of I. Walker Hall's work on the Purins than of any other.

---

**Sexual Impotence.** By Victor G. Vecki, M. D., Consulting Genito-Urinary Surgeon to the Mt. Zion Hospital, San Francisco, Fifth edition, enlarged. 12mo. of 405 pages.

Philadelphia and London: W. B. Saunders Company, 1915. Cloth, $2.25 net.

This work has become standard by its previous editions. It is full without being prolix, indeed the number of apparently extrinsic factors which have a bearing on the subject, is surprising. So far as possible, the author deals with the subject from an unbiased medical standpoint, although it is obviously impossible to avoid moral and social features.

---

**Venereal Diseases.** A Manual for Students and Practitioners. By James R. Hayden, M. D., F. A. C. S., Professor of Urology at the College of Physicians and Surgeons, Columbia University, New York; Visiting Genito-Urinary Surgeon to Bellevue Hospital; Consulting Genito-Urinary Surgeon to St. Joseph's Hospital, Yonkers, New York. 12mo., 365 pages, with 133 illustrations. Cloth, $2.50 net. Lea & Febiger, Publishers, Philadelphia and New York, 1916.

The aim of the author is distinctly practical. It is easier to describe the work in a negative than in a positive way. Imagine that one eliminates the detail incident to a dermatologic and neurologic consideration of syphilis, that he is not a surgeon and not especially interested in any given highly technical instrumental procedure, as ureteral catheterization or cystoscopy, that he is not an amynologist nor a sociologist. He would then, with a full understanding of anatomy, physiology and pathology and a desire to write a thorough treatise on venereal diseases, with due attention to diagnosis, prophylaxis and therapeutics, write just about such a text book as the present. We do not mean to imply that the details mentioned have been altogether ignored, merely that they have been reduced to a practical working basis.

